# Case Study in Youth Flexible Assertive Community Treatment: An Illustration of the Need for Integrated Care

**DOI:** 10.3389/fpsyt.2022.903523

**Published:** 2022-05-12

**Authors:** Marieke Broersen, Nynke Frieswijk, Rob Coolen, Daan H. M. Creemers, Hans Kroon

**Affiliations:** ^1^GGZ Oost Brabant, Oss, Netherlands; ^2^Tranzo – Tilburg School of Social and Behavioral Sciences, Tilburg University, Tilburg, Netherlands; ^3^Accare, Groningen, Netherlands; ^4^Trimbos Institute, Utrecht, Netherlands

**Keywords:** Flexible Assertive Community Treatment, mental healthcare model, adolescents, early intervention, family centred, integrated care approach, fragmented care, intensive case management

## Abstract

Youth Flexible Assertive Community Treatment (Youth Flexible ACT) is a client- and family-centered service delivery model for young people up to 24 years of age who have interrelated psychiatric- and social problems across multiple life domains and do not readily engage with office-based mental health services. Youth Flexible ACT teams were set up to meet the multifaceted needs of this subgroup in an integrated manner. In this paper, we present a case study to (1) describe the core principles of Youth Flexible ACT and (2) illustrate the application of the mental healthcare model. Subsequently, we describe the contribution of model elements to a positive care process and discuss the challenges of the team in working with the model. The case study displays the importance of integrated flexible and personalized care services to enable adolescents to remain engaged in care.

## Introduction

Michelle (20 years) is a well-groomed young lady with long brown hair. She is lying on a large comfortable couch at home, having the television on in the background. She is bored and busy with her phone. She would like to go shopping to buy herself a new shirt, but she doesn’t dare to go out on the streets alone. She lights up another cigarette. Michelle has been suffering from panic attacks for a while. She hasn’t been going to school for a few years now, and she doesn’t have a work certificate. Her friendships are diluted. She lives with her ex-stepfather and deals with stressful family situations. Michelle has been receiving outpatient mental health care services for years now, without lasting results. Recently, caregivers from a Youth Flexible Assertive Community Team (Youth Flexible ACT) have started visiting her at home.

Like Michelle, some children and adolescents face difficulties in multiple areas of daily life due to psychiatric- and comorbid problems, such as family stress situations, substance misuse, and/or problems with intellectual functioning (1–4). Frequent everyday difficulties include problems with attending school, finding and keeping a job, peer relationships, housing, the legal system or police, and/or personal finance. These children and adolescents are often raised in families coping with psychiatric, financial, addiction, or parenting and relationship problems (1, 5). In short, these young people require multiple types of care in several domains.

The secondary outpatient (office-based) mental health care does not seem to be able to effectively meet the care demands of these adolescents due to the risk of fragmentation of services, the silos in service provision, and thereby treatment discontinuity and dropout (6–9). In addition, many adolescents of this subgroup already have a history of (secondary) mental healthcare. They have difficulty trusting services, or they have grown tired of them. Moreover, these young people are in a vulnerable developmental stage as they undergo the transition from adolescence to adulthood, a time with many key transitions in their lives. Transitional age youth are at risk of falling through the cracks of the system designed to provide Child and Adolescent Mental Health Services (CAMHS) or Adult Mental Health Services (AMHS), often not considering the specific needs of these transitional generation (10).

Michelle had to deal with sexual abuse by a family member, domestic violence, and ever-changing household compositions from a very young age. The local child welfare system supervised her family. At the age of 8, Michelle received play therapy at a small psychology practice to learn to cope with emotions, feel more secure, and be more assertive. An intelligence test was then administered, revealing average cognitive abilities. At 10, the therapy ended, as Michelle seemed to feel better about herself.

When she was 15 years old, Michelle refused to go to school. She was then referred to a mental health care institute by the local public health service. There, she was diagnosed with Post-Traumatic Stress Disorder (PTSD) for which she received intensive trauma treatment. Her trauma symptoms (i.e., nightmares where she re-experienced the sexual abuse) had successfully diminished. Michelle and her family were then advised to follow Systemic Family Therapy (SFT) due to the difficult relationships within the family. Yet, SFT was not desirable nor deemed feasible by Michelle and her family. The case was closed by mutual consent. Her family was still under the supervision of the local child welfare system.

At the age of 17, Michelle was referred to a mental health care institute by her general practitioner because of recurring PTSD symptoms, which she suffered since a human trafficker threatened her. She also had panic attacks, mood swings, and became underweight. Meanwhile, she did not undertake activities independently and still refused to go to school. Eye Movement Desensitization and Reprocessing therapy (EMDR; to treat traumatic stress symptoms), Psychomotor Therapy (PMT; to improve self-esteem and assertiveness) and Psychiatric Intensive Homecare (PIH; to provide support in the home situation to generalize age-related developmental tasks in the home environment) were started but did not continue. The EMDR treatment did not go smoothly, as she often missed her appointments; likewise, PIH was discontinued due to non-compliance with appointments. It was too difficult for Michelle to go to the PMT sessions. She had little trust in the male therapist. The treatment was closed, and the head psychologist reported her concerns about the worrisome wellbeing of Michelle and her siblings to Child Protective Services.

At 18, Michelle was re-referred to the outpatient (office-based) department of a mental health care organization for her PTSD symptoms and panic disorder with agoraphobia. Michelle struggled with various psychiatric and social difficulties in multiple areas of her everyday life: she had trouble going to school, financial problems, family system problems, and difficulties getting along with peers. Due to the complexity of the problems, an extensive psychological diagnostic assessment was started to give direction to treatment. Psychologists noted that Michelle’s avoidant and dependent attitude threatened her personality development. However, the diagnostic process could not be performed successfully due to several missed appointments. Michelle kept her distance from mental health workers. Michelle was then referred to the Youth Flexible ACT team of the same mental health care organization.

Michelle’s mental healthcare history illustrates that it is hardly possible to set up a solid care process in traditional office-based mental health care for adolescents with multifaceted care needs without an integrative, contextual, and outreaching approach. Appropriate care is urgently needed to manage symptoms and complaints, to move forward in age-related developmental tasks, and, in the long term, to prevent adverse outcomes in adulthood (11, 12). Fortunately, the number of youth integrated intensive mental health care approaches has been increasing globally (13–16), of which Youth Flexible ACT is an example. Flexible ACT is a Dutch adaptation and elaboration of Assertive Community Treatment (ACT) (17). This client-centered service delivery model integrates evidence-based practices and treatments and follows the multidisciplinary guidelines to support clients in their symptomatic, personal, and social recovery. Adult Flexible ACT is the standard service delivery model for people with severe mental illness in the Netherlands, with ∼400 Flexible ACT teams operational (18). In addition, the model is adopted in various other countries (19–23). The Adult Flexible ACT model is adapted to young people (0–24 years of age) who experience interrelated psychiatric- and social problems and do not readily engage with office-based traditional mental health services (intake criteria; Table 1) (1, 2). Youth Flexible ACT is becoming more widespread in the Netherlands, with ∼60 teams being active or under development (18). In addition, the model is gaining international appraisal with the first teams being implemented in Norway. Although the popularity of Youth Flexible ACT is increasing, evidence supporting the effectiveness of this particular model remains scarce. Therefore, our ongoing Multicenter Youth Flexible ACT Study examines the effect of Youth Flexible ACT on a group of 199 adolescents (24). Yet, a clear description of the model with its core principles has not been described before in the international literature. This is the first article to make it widely available. In this paper, we present a case study to (1) describe the core principles of Youth Flexible ACT and (2) illustrate the application of the mental healthcare model. Subsequently, we describe the contribution of model elements to a positive care process and discuss the challenges of the team in working with the model.

**TABLE 1:** Youth Flexible ACT intake criteria.

Criterion 1 Age-range	The client is ≤22 years of age.
Criterion 2 Mental health disorder	The client is diagnosed with a mental health disorder (or presumptive diagnosis) for which multidisciplinary treatment is required.
Criterion 3 Difficult to engage	The client cannot successfully attend office-based treatment due to the complexity of mental illness or by actively refusing contact.
Criterion 4 Case complexity	The client’s situation is perceived as complex and problematic (e.g., risk of self-neglect, psychiatric decompensation, suicide, domestic violence, child abuse, or self-harm) with insufficiently protected factors (e.g., adequate coping, employment or daily structural activities and a support system). The client experiences difficulties in multiple areas of daily life (e.g., problems with education, employment, peer relationships, housing, finances, health, substance abuse and issues with the criminal justice system) that require multi-agency involvement.
Criterion 5 Family issues	The client and/or family face family system problems and/or parenting issues.
Criterion 6 District	The client lives in the district of the Youth Flexible ACT team.

## Youth Flexible ACT in Practice

Flexible ACT teams are multidisciplinary teams providing long-term assertive outreach care in a flexible and integrated manner. These teams deliver a complete range of services on a continuum of care, including treatment for psychiatric symptoms, practical support with daily living needs, and rehabilitation and recovery support (25). Flexible ACT teams set up a collaborative effort with clients, their families, friends, and other key support figures to activate the network around someone. The Flexible ACT model is adapted for several target groups, including adults, youth, forensic clients with psychiatric difficulties, and people with mild intellectual disabilities. Core principles of Flexible ACT are integrated care, network-oriented and coordinated care, flexible stepped-up care, staging of care, and accessible, assertive, and outreach care, thereby providing high-quality and personalized care (Table 2) (25, 26).

**TABLE 2:** Core principles of Flexible ACT.

**1. Integrated care** Flexible ACT teams incorporate a holistic focused care approach, providing care across several domains, including psychiatric, addiction, and supportive care. The treatment and support are integrated by a multidisciplinary team that works closely together and often encounters each other in regular team meetings (e.g., daily morning meeting, weekly treatment plan meeting, consultation between colleagues) and shared office space. Teams consist of various professionals, including a psychiatrist, psychiatric nurses, psychologists, social workers, a peer support worker, an employment specialist, and an addiction specialist. Expertise in somatic care, intellectual disability, and judicial support is available. The peer support worker has an important role in self-direction, recovery, and combating (self-) stigmatization and provides individual contacts and recovery courses, such as “Do It Yourself Recovery” and “Wellness Recovery Action Plan.” In addition, sports activities can be organized, such as running therapy or gym activities.
**2. Network-oriented and coordinated care** As a single team cannot provide all services, professionals from other healthcare organizations and social services are engaged when necessary (e.g., from secondary office-based treatments, primary care, inpatient clinics, crisis service for mental health, social work, employers, providers of organized daytime activities, assisted living accommodations, schools, the municipality). Flexible ACT teams collaborate closely with these other services in the district. Good connections with network partners also ensure a “smooth” transfer when clients exit Youth Flexible ACT and still need assistance from other care services. Teams also serve a directing and coordinating function to retain the overview of the client’s treatment and the frequently fragmented care. In addition, Flexible ACT teams try to establish close contacts with the informal network to empower the client and close contacts as much as possible. For example, the informal network is involved in the intake phase, psychoeducation, setting up a crisis and contingency plan, and systemic interventions.
**3. Flexible stepped-up care** Flexible ACT teams deliver two modes of care: individual case management and intensive team care. The intensity of care can be stepped up or down, as needed. Temporary care may be intensified, e.g., during an (imminent) crisis, decompensation after discharge from a hospital, or in the event of major positive or negative life events. Clients receiving individual care have a case manager and a head practitioner (psychiatrist, healthcare, or clinical psychologist). Other team members can also be involved when required to address specific elements of treatment or support. A client who needs intensive team care will receive care from several team members (shared caseload). During the daily digital board team meeting, all clients receiving intensive team care will be discussed briefly to determine which interventions will be applied, which relatives of the client will be involved, and which team members will deliver the intervention. Network partners can also be involved in the care intensification. The flexibility and stability of stepped-up care ensure that clients can engage with the same team during the different phases of the recovery process. Unplanned psychiatric inpatient admissions are therefore limited as much as possible. Yet, scheduled psychiatric inpatient admissions can still be part of the treatment as care providers remain involved and visit the client at the psychiatric inpatient clinic. Using the daily digital board meeting, teams can share caseloads. Because team members are aware of the cases and participate in the decision-making about the goals and interventions, every team member can assist when needed.
**4. Staging of care** As every young person follows an individual recovery path, it is important to identify the current stage(s) of recovery and behavioral change in which the client is situated and provide stage-matched interventions. In this way, the care is continuously tailored to the client’s needs, the clients’ control is maximized, and a trusted working relationship with shared mutual goals and appropriate treatment expectations is supported. Generally, clients move through different stages of change, as described by the Stages of Change Model developed by Prochaska and DiClemente (32): (1) pre-contemplation (no intention to changing behavior), (2) contemplation (aware of problems exists but not committed to change), (3) preparation (intends to take action to change), (4) action (executing a plan to modify behavior), (5) maintenance (sustaining changes and developing new behavior, relapse prevention plan). The process of change and recovery is dynamic and continuous and does not entail a rigid sequence of stages. A client can also be in different stages for several goals simultaneously. This dynamic character is reflected in the client’s goals and treatment plans in which recovery is present during all stages. Adolescents who are skeptical or unsure about the potential of Youth Flexible ACT (precontemplation stage) may agree to explore how the team could help them instead of starting interventions directly. In line with the previous, the mental health workers’ actions can also be divided into stages (1) initiating the contact (see the person and not only the problem), (2) sustaining contact (explore clients’ wishes and burdens), (3) working together on developmental tasks and recovery (providing diagnostic assessments, psychological interventions, school support, etc.), (4) phasing out Youth Flexible ACT interference gradually (when a client shows consistent improvements).
**5. Accessible, Assertive, and Outreach care** The support system can be involved more easily, and learned skills and insights can be applied immediately through outreach care. In addition, the flexible ACT teams act in an assertive way, actively reaching out to both clients and other (healthcare) organizations to maximally engage clients in care. Team members often visit clients at their homes or other preferred locations. Repeated no-shows or non-compliance with agreements are reasons for a conversation or an unexpected visit without blaming or criticizing. Furthermore, teams aim to be approachable and accessible. For example, team members are easy to reach *via* mobile phone, and some teams also provide Flexible ACT in the evening hours and on the weekend.
**6. Model fidelity** Flexible ACT teams are expected to work according to the Flexible ACT model’s guidelines. The fidelity guideline stipulates that the team must deliver the most appropriate services, interventions, and actions to their target group. The Centre for Certification ACT and Flexible ACT (CCAF) (CCAF workbook) developed a model fidelity scale based on the flexible ACT guidelines to determine how teams adhere to the model. According to the guidelines, a high model fidelity score indicates that the team provides both multidisciplinary treatment options and optimal care support. Model fidelity scores are determined through audits. Multiple scales exist for different target groups (adults, youth, forensic, and intellectual disability). Flexible ACT is continuously refined based on experiences and the latest scientific insights. The teams are expected to provide high-quality care (following clinical practice guidelines and evidence-based practices). A client panel is organized annually in which clients provide feedback on the team’s functioning. The collaboration with other service providers is evaluated in an annual network meeting.

Youth Flexible ACT, in which the family system plays a major part, has important additional features beyond Adult Flexible ACT. Youth Flexible ACT addresses the age-related developmental needs of both children and young adults. Specifically, identity formation is central in adolescence, and care providers need to support the following developmental tasks: shaping changing relationships within the family (moving from dependence to autonomy), stimulating contact with peers (peers become more important as a reference group as the influence of parents decreases), participating in education or work and filling leisure time (1, 5, 27). For these reasons, Youth Flexible ACT, unlike Adult Flexible ACT, includes also a systemic family therapist, an employment and education specialist, and parent- and family counselors are additional team members. In essence, Youth Flexible ACT team members have a hopeful attitude: thinking in possibilities, thinking along with the client, thinking about strengthening personal capabilities, focusing on self-direction, and the use of positive language.

### Initial Phase: Intake at Youth Flexible ACT Team

The standard intake contains three interviews and an advisory conversation with clients and their relatives. Yet, the number of interviews can vary according to the requirements of a joint treatment plan. The interviews can be conducted at the office or alternative locations of clients’ choice. Time is devoted to building and maintaining a relationship of trust between mental health workers, clients, and their relatives. This is necessary, as clients are often not ready to receive care services. The interviews with both the client and family members discuss the benefits of psychiatric treatment. Michelle was referred to the Youth Flexible ACT team internally by the outpatient (office-based) department, as they did not succeed to start a care program sufficiently. Michelle said: “I’m open to support, but I don’t think it will work. I’ve had so much support before; it’s hard to think it is any different now.” Michelle tended to avoid appointments for the intake interviews, as she did not feel comfortable in previous care. Her ex-stepfather indicated that Michelle could start to avoid care when certain questions or treatment become too personal. In addition, Michelle indicated that it was difficult for her to get to appointments because she had to arrange for her ex-stepfather or mother to drive her. To overcome Michelle’s avoidance, it was agreed that the interviewers would take an open and transparent attitude and that they would follow her pace. This enabled Michelle to feel relaxed when meeting with the care providers and allowed the interviewers to collect the necessary intake information. Because Michelle indicated that she had a lot on her mind, a Psychiatric Nurse Practitioner (PNP) started to visit her immediately twice a week to provide support during the intake phase.

**FIGURE 1:**
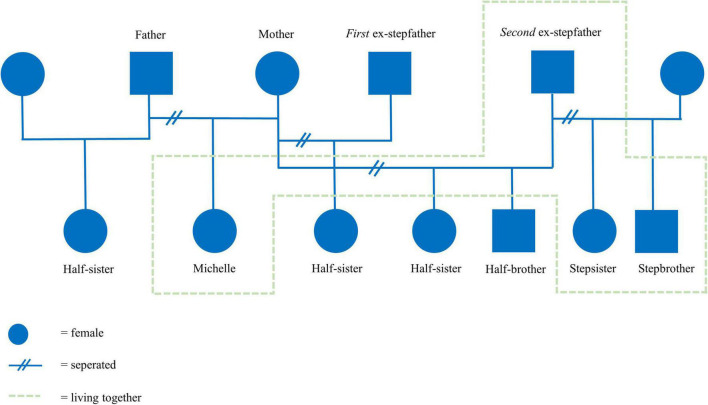
Family genogram.

**FIGURE 2:**
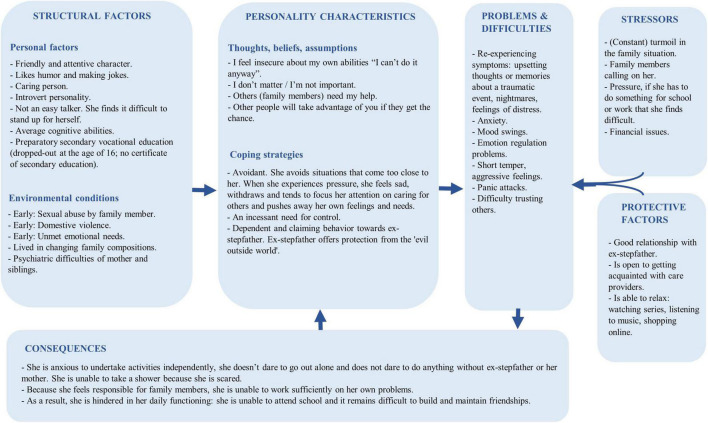
Holistic problem analysis (based on a holistic model from the Dutch Association for Behavioural and Cognitive Therapies).

After 3 months, the intake process was completed successfully, yielding the following diagnostic summary. An 18-year-old woman was referred to the treatment due to ongoing trauma, panic attacks, school dropout, and a difficult family situation. She had experienced multiple traumatic events (sexual and abusive) that have had a major influence on her self-image and image of others. Consequently, Michelle showed avoidant coping behavior in difficult situations, poor coping skills, and anger. She had to deal with several family issues at home and had gone through many changing family compositions (Figure 1). Michelle’s symptoms were classified as PTSD and agoraphobia. A hypothesis about the coherence of Michelle’s problems was formulated (Figure 2). Subsequently, a treatment plan that described treatment goals was drawn with Michelle (Table 3).

**TABLE 3:** Michelle’s treatment goals.

Care process	- I’m willing to give Youth Flexible ACT a try, although I was disappointed in previous mental health care. I have appointments with the Flexible ACT mental health workers, and I got to know them, and they got to know me (and my family system). - I understand why previous care has not been successful.
Mental health	- I understand why I’m afraid to undertake activities (i.e., school, going to the store) without my ex-stepfather. - I want to be less anxious in public spaces and dare to go out alone again. - I want to deal with my emotions more adequately. When I feel aggression coming on, I know what to do to deal with it. - I suffer less from re-experiences and nightmares. - I have a mental health crisis and contingency plan, so my support system and I know what to do when an emergency arises.
Education/employment	- I’m going to school or have a job.
Family and relationships	- I’m aware of my own needs, and I want to obtain a healthy balance between my needs and the care for others. - I want better communication with my mother and ex-stepfather. I often feel misunderstood.
Finance	- I want to oversee my financial situation.

#### Routine Outcome Monitoring

As part of Youth Flexible ACT involvement, baseline and follow-up outcome measures were administered to assess overall psychological distress (OQ-45) (28, 29) and severity of PTSD symptoms (PSS-SR) (30, 31). The OQ-45 is a self-report questionnaire consisting of 45 items yielding a total score and 3 subscale scores. Items were scored on a 5-point rating scale, ranging from never (0) to almost always (4). A cut-off score of 55 on the full scale indicates clinically significant distress (28). The PSS self-report version consists of 17 items corresponding to the PTSS symptoms listed in the Diagnostic and Statistical Manual of Mental Disorders, Fourth Edition (DSM-IV) (31). Items were scored according to the frequency of symptom occurrence in the past week, ranging from 0 (not at all) to 3 (5 or more times per week). The PSS-SR provides a total score and three subscores. Higher scores represent more severe symptoms.

### Middle Phase: Active Treatment

#### First-Year of Flexible ACT Care

After the intake report and treatment plan had been approved, the active treatment phase could start. Multiple team members were assigned to Michelle:

1) A head practitioner (healthcare psychologist/skilled in systemic working) coordinated the care process, became acquainted with the family system, mapped out why previous care services failed and provided EMDR therapy;
2) A PNP provided support to help Michelle cope with problems in her everyday life and provided parent counseling sessions;
3) A social worker (also skilled in systemic working) provided Emotion Regulation Therapy (ERT) and offered support with searching and referring to community resources, such as school or job opportunities, and with conducting parent counseling sessions, and
4) A psychiatrist examined whether medication could be a helpful addition.

Official systemic therapy was not indicated, as team members believed therapy would not be feasible for the family due to the stressful and changing home situation. Additionally, immediate treatment for trauma and anxiety symptoms was not indicated due to Michelle’s limited trust in mental healthcare workers.

##### Care Process

During this first year, the team focused on initiating and sustaining contact with Michelle and her ex-stepfather. Gradually, the involved team members got to know her better, and Michelle started to speak freely. Michelle had gained more confidence in the care process.

##### Mental Health

Michelle explained to the PNP that she was afraid to take a shower and go to the dentist. When showering, she experienced a lot of tension because of earlier traumatic events in the bathroom. The PNP practiced exposure therapy techniques with Michelle to become less anxious to go out alone and supported her with visits to the dentist. Additionally, they started setting up a crisis and contingency plan. Meanwhile, the social worker started with ERT, and during the year, Michelle gained more insight into her emotional self-regulation. Furthermore, Michelle started taking fluoxetine to feel calmer, less panicky, and less agitated. When necessary, she also took quetiapine to suppress her unrest.

At the end of the year, Michelle managed to undertake activities by herself (go to the store, dentist, shower). She appeared calmer and indicated she would like to start trauma therapy.

##### Education/Employment

With the support of the social worker, Michelle set up a resume and dared to take the step to apply for work in a department store. The social worker had arranged that Michelle could start working while she continued to receive disability benefits. Michelle was hired, and the employer was very helpful and understanding of her mental health issues. The first working days went well, and Michelle was very proud.

##### Family and Relationships

Psychiatric Nurse Practitioner provided psychoeducation to the ex-stepfather, Michelle’s mother, and her therapist, explaining Michelle’s problems and describing the ways in which her parents could support her. Michelle has managed to distance herself from the home situation during the year by focusing on the application process and work. However, the team members noticed that Michelle was still very dependent on her ex-stepfather. Although her situation had improved, she hardly ever left his side.

##### Finance

Together with the social worker, Michelle took steps in administrative matters, managing her financial situation.

#### Second-Year of Flexible ACT Care

Eye Movement Desensitization and Reprocessing therapy sessions were scheduled in the second year. The conversations with the PNP, social worker, and psychiatrist continued.

##### Care Process

Michelle gained more insights into her avoidance pattern during the sessions with the involved team members. She understood the role of avoidant coping and distrusting others in previous (unsuccessful) treatments.

##### Mental Health

During the introductory talks with the EMDR therapist, Michelle pointed out that she had not yet reported sexual abuse to the police and wanted to do so. They decided first to file a report and subsequently start EMDR. However, when the EMDR sessions eventually started, she kept canceling the sessions. She mentioned that she had a lot on her mind concerning her home situation. Her half-sister was in a closed youth care institution, and Michelle was very worried about her. Furthermore, her mother lived temporarily with Michelle’s ex-stepfather for her mother’s safety. This caused a lot of stress in the family, eventually leading to Michelle’s emotional outburst. Michelle took (small) overdoses of sleeping pills to suppress these emotions. The team members who were already involved were increasing the contact frequency. Other team members knew what was going on and, when necessary, could help immediately. After 2 weeks, when Michelle was calmer again, the situation was re-evaluated with Michelle and family members and with her mother’s therapist. Together with the social worker, Michelle talked about her stress triggers, methods she could use to manage and reduce stress symptoms, and different ways she could ask for support.

##### Education/Employment

Michelle had had a good start at work and handled her fears. She understood that she had not gained successful and positive experiences due to anxiety and avoidance, which hurt her self-image. Now, she showed perseverance. Unfortunately, after a few months, she suffered from tension headaches and experienced a lot of stress due to family issues. Michelle couldn’t handle going to work, and as a result, her temporary contract was not being renewed.

##### Family and Relationships

Michelle, her mother, and her ex-stepfather knew that Michelle had to maintain a healthy balance between paying attention to others and doing what was good for herself. Acting on this has proven difficult. Her mother and her mothers’ therapist discussed that the mother should not take responsibility for Michelle.

##### Finance

Michelle missed an appointment with the occupational physician, for which a fee was charged. Michelle was already stressed, and then experienced even more stress as she couldn’t afford this. She told the social worker she also failed to pay her healthcare insurance. Eventually, the income and work consultant of the municipality had set up a plan through which a payment arrangement was agreed. Michelle knew she should have contacted several organizations to arrange her finances but did not do so. She found it very difficult to make the necessary phone calls, and because she felt ashamed, she postponed and avoided this. The social worker discussed and addressed this recurring avoidance tendency with Michelle.

#### Third-Year of Flexible ACT Care

During the third year, an IPS counselor (Individual Placement and Support) was deployed to help Michelle search for work. The EMDR sessions were continued, and the social worker still provided support and parent counseling sessions. Medication continued to be administered. The PNP was only deployed when necessary.

##### Care Process

The EMDR sessions, during which the social worker was closely involved by driving Michelle to and from the sessions and providing aftercare during the supportive contacts, were resumed. This agreement gave Michelle a safe feeling and helped her deal with emotions in daily practice.

##### Mental Health

Michelle no longer suffered from trauma-related complaints. She had no traumatic memories, no nightmares, and she was less hyper-alert. Michelle felt less irritation and anger and dealt with her emotions more adequately. Because her level of tension decreased, she was much more daring to go out alone and less dependent on her ex-stepfather. She even got her driver’s license.

##### Education/Employment

Michelle was introduced to the IPS counselor, and together they set up a new work plan. A basic qualification was required to retain social assistance benefits for a good starting position in the labor market. Michelle refused to take classroom education, and given her avoidance pattern, it was not expected to be successful at this time. A home study (preparation for maternity nurse) was set up to get her used to education. The IPS counselor contacted the municipality, and they paid for the home study and laptop while retaining sickness benefits. Michelle was afraid to start with the study, but it did go well after a few days. However, after a few months, Michelle had trouble following the study. She indicated that the discussed theories were too difficult to understand and gave her a feeling of failure. Initially, Michelle wished to quit her study, but after joint meetings with the social worker, Michelle, and other relatives, she managed to catch up with the study again.

##### Family and Relationships

Michelle was much less irritated and more patient with her relatives. She was more successful in making choices for herself (going to therapy, studying) and stopped trying to take over someone else’s problems. For example, when her mother moved to another house and called on her for help, Michelle could cope in a controlled and calm way.

##### Finance

At the request of Michelle, her ex-stepfather helped her manage financial matters.

#### Fourth-Year of Flexible ACT Care

During the fourth year, the spread of coronavirus caused lockdowns worldwide. In the beginning, sessions mainly took place by telephone or video calling, in the garden, or during walks. IPS, EMDR, sessions with the social worker, and medication consults were continued.

##### Care Process

Halfway through the year, a horrible event took place. Michelle’s half-sister, still a minor, went missing. Luckily, her sister returned home after 2 weeks. During this period, the team care provided additional support, and a social worker, the PNP, or head psychologist visited Michelle daily.

##### Mental Health

Because of the episode with her sister, new cases of abuse came to light that triggered trauma complaints. The EMDR sessions were resumed with images that were not treated before. The sessions went well. Michelle still experienced some anger but indicated she was no longer re-experiencing the symptoms. During the fourth year, Michelle showed much more control in handling her emotions. Despite the turmoil in her family, Michelle was more stable and started to cope better with the things she’s gone through. She could handle setbacks more easily and react more calmly. Her medication (fluoxetine) was gradually reduced.

##### Education/Employment

Michelle caught up with her studies but soon indicated that it took a lot of effort for her to consistently comprehend the theory and that she wanted to stop with the study. The IPS counselor listened and indicated that a work-study program suited Michelle better. Individual internal training at an organization seemed more appropriate than classroom training or home study. Together with the IPS counselor, Michelle had visited several employers, and she applied for a learn/work trajectory at a healthcare institution.

##### Family and Relationships

During the fourth year, Michelle developed better communication with family members and her mother and ex-stepfather did no longer involve her as much in their daily struggles. Michelle managed to maintain a healthier balance between her needs and those of others. She had more control in taking care of things for herself (i.e., reschedule an appointment, make a phone call to institutions). When Michelle’s two youngest siblings came to live with her ex-stepfather, it was getting more crowded, and Michelle expressed carefully that she needed a place to live on her own. The team already had this in mind earlier but deliberately did not focus on it because they still expected resistance and avoidance at that time. The social worker helped Michelle explore other housing options.

### Final Phase: Exiting Youth Flexible ACT

Flexible ACT care is provided as long as necessary and as briefly as possible to achieve symptomatic, functional, and personal recovery. Whether the specialized intensive mental health care can be downgraded to primary [General Practitioner (GP), primary care psychologist, social district team] or secondary office-based mental health care is continuously monitored throughout the care period and carefully considered by taking into account symptomatic remission, complex medication use, school attendance, having a job or other form of daytime activities, availability of support systems, financial situation, confidence in the recovery, and ability to accept guidance and call for help when necessary. In addition to leaving care upon recovery, clients exit Youth Flexible ACT when they reach the age limit. When clients reach the age of 23, various forms of follow-up care are considered, depending on the situation. Exits and transfers are discussed and planned carefully with the clients and their relatives and are structured and staged. The team ensures a “smooth” transfer from old to new care providers through shared contacts to guarantee continuity of care.

Michelle recently turned 23; therefore, the Youth Flexible ACT team discussed with her the transition to AMHS in the coming year. Michelle and the team members agreed that intensive Flexible ACT care was no longer necessary While Michelle reported high scores on overall psychological distress (OQ-45) and on severity of PTSD symptom (PSS-SR) at the start of Flexible ACT, her distress and trauma symptoms decreased sufficiently at the end of Flexible ACT (Table 4). Michelle still experienced some anger after the EMDR sessions, but she was no longer suffering from re-experiencing symptoms. The communication with family members improved. She applied for a work-study program, felt more empowered, and made choices and arranged things for herself.

**TABLE 4:** Overview of baseline and follow−up results.

	Range	Baseline	Follow-up 1	Follow-up 2	Follow-up 3	Follow-up 4
		Enter F-ACT	During F-ACT	During F-ACT	During F-ACT	Exit F-ACT
**OQ-45**						
Total score	0–180	80	67	102	57	32
Symptom distress	0–100	55	44	75	35	25
Interpersonal relations	0–44	18	17	17	13	4
Social role	0–36	7	6	10	9	3

		**Pre-EMDR**	**Post-EMDR**	**New EMDR**	**New EMDR**	**Post-EMDR**

**PSS-SR**						
Total score	0–51	42	0	8	11	11
Re-experiencing	0–15	12	0	4	7	7
Avoidance	0–21	16	0	2	1	0
Hyperarousal	0–15	14	0	2	3	4

*F-ACT, Flexible Assertive Community Treatment; EMDR, Eye Movement Desensitization and Reprocessing; OQ-45, Outcome Questionnaire 45; PSS-SR, Post-Traumatic Stress Disorder Symptom Scale – Self-Report.*

The final phase (*phasing out*) would focus on preparing for the transition to adult- and primary care, maintaining the progress achieved, and preventing Michelle from reverting to negative behavior. Setting up a relapse prevention plan, which describes triggers, coping tools, and support group information is always an important part of this final phase. The following interventions were included in the final treatment plan of Michelle: setting up a relapse prevention plan, continuing IPS counseling, transferring medication to a general practitioner, contacting mother’s therapist, contacting involved social services, and becoming acquainted with a new mental health care provider. The IPS trajectory could be continued without Flexible ACT.

## Discussion

We selected the case of Michelle for description in the present article as it aptly demonstrates the need for integrated care across different domains, which is at the core of Youth Flexible ACT. Her age was above 18, she had problems in multiple life domains and she had a long history in care, all of which are hallmarks of the Youth Flexible ACT population (2). Yet, it should be noted that this case is not meant to serve as a representative client, because such a client does not exist due to the heterogeneous nature of the care models’ target population. In fact, we previously showed that this population consists of four subgroups: the “internalizing,” “externalizing,” “non-specific,” and the “overly impulsive” subgroup (2). Michelle’s case best matches the “externalizing” subgroup, characterized by emotion regulation problems that manifest in disruptive behavior. In addition, the case of Michelle distinguished itself from other cases by the complicated family relationships and -situation in which stressful life events occurred frequently, and by multiple family members being in (Flexible ACT) care. As a consequence, a lot of the teams’ time was devoted to collaboration and coordination with various health organizations and family members.

### Overview of the Care Process

Michelle remained in care and eventually completed the full trauma treatment. Michelle’s mother and ex-stepfather saw Michelle happy again and indicated improved communication at home. Michelle told the Youth Flexible ACT team members that she finally felt heard and understood. Team members had an open-minded and non-judgmental attitude and wanted to listen to her complaints, no matter how angry she was. Team members pointed out that the positive nature of this care process was largely due to the close collaboration between team members, open communication in the team, and the expressed awareness and appreciation for each other’s contribution, fostered by the regular meetings that are part of Flexible ACT (such as the daily team meeting). It should be noted, however, that it is impossible to ascertain the degree to which Michelle’s natural development (given her stage of life) contributed to the improvements she made over the course of the follow-up period. Yet, we argue that Youth Flexible ACT enabled Michelle to take the age-related developmental steps that are part of her natural development. In fact, this was not a given due to the multitude of problems she faced combined with her difficulty to engage these problems through regular care.

While no single model element is individually accountable for positive results, the combination of model elements contributed to the successful outcome:

#### Integrated Care

Michelle had to deal with multiple difficulties in everyday life, including problems with mental health, school and work attendance, family issues, and financial situation. A multidisciplinary team tackled these problems across several care domains in an integrated manner. The team’s integrated and multidisciplinary nature enabled Michelle to stay engaged in care and accomplish treatment according to the clinical guidelines. For example, in addition to EMDR therapy, the continued use of (practical) supportive contacts helped Michelle deal with acute stressful life events and remain sufficiently calm and focused during EMDR therapy.

#### Network-Oriented and Coordinated Care

The Flexible ACT team worked contextually and systemically. The team collaborated and coordinated with other involved service providers (i.e., service provider for mother, municipality, employers). In addition, team members established close contacts with the informal network (family members) and involved them in treatment when required. For instance, the ex-stepfather was often present during conversations, and team members also had regular contact with her mother (and mother’s therapists). The team learned about the family’s rules, values, family language, and behavioral patterns.

#### Flexible Stepped-Up Care

Flexible ACT teams deliver two modes of care: individual case management and intensive team care. When Michelle felt a lot of stress, the team could quickly intensify the care. Michelle received care from several team members (shared caseload), and her situation was discussed in the daily digital board meeting. The healthcare psychologist, social worker, or the PNP visited her daily. They monitored her situation and taught her emotion regulation techniques that helped her evaluate each situation objectively. Her mother and ex-stepfather were involved, and the intensification of care was evaluated with all involved to facilitate handling a crisis in the client’s environment and prevent further psychiatric admission.

#### Staging of Care

Care trajectories within Youth Flexible ACT often do not have a steady course. Instead, the haphazard nature of such treatment trajectories was also reflected in Michelle’s case. The Flexible ACT team provided stage-matched interventions, thereby utilizing the Stages of Change Model (Table 2) (32). Michelle (1) was given the time to get used to and trust the Flexible ACT team members, allowing her to feel comfortable to receive the treatment (pre-contemplation), (2) considered EMDR treatment but postponed it first (contemplation and preparation), (3) was actively engaged in treatment (action), and (4) attempted to consolidate the new helping behaviors she experienced during treatment and prevented relapse (maintenance).

Initially, Flexible ACT focuses on improving the client’s engagement according to the client’s wishes, consequently reducing the risk of treatment dropout. Michelle and her clinicians built a healthy working alliance based on non-judgment and transparency. Moreover, Michelle’s own needs were addressed right at the start. Instead of focusing on reducing PTSD symptoms, Michelle first received help managing her financial situation and drafting a curriculum vitae before starting EMDR therapy. This way, Michelle felt understood and had been given a choice. Immediate treatment for trauma and anxiety symptoms would not have been feasible due to little trust and difficulty to speak freely.

#### Accessible, Assertive, and Outreach Care

The team’s easily accessible, strong, and outreaching character helped Michelle stay engaged in therapy. Michelle missed quite a few therapy sessions, but progress was maintained and continued due to the team’s easy accessibility (use of work phones), flexibility to schedule sessions, and directive and coordinated approach of team members. Moreover, it was often difficult for Michelle to arrange transport to the office, which was addressed by the team’s ability to meet at home or other preferred locations or pick her up and bring her back home.

### Challenges in Working With the Model

The Flexible ACT team encountered challenges in working with the service model. Specifically, it was challenging to (1) integrate evidence-based interventions systematically within everyday practice, and (2) end Youth Flexible ACT care because of the age limit. We describe potential ways to address these challenges.

First, the challenge for the team was to apply disorder-specific guidelines and standards of care systematically in a hectic care environment that is characterized by daily unexpected events. The case manager therefore plays an important role in guarding the treatment process from haphazard distractions, especially during daily team meetings and periodically planned treatment evaluations. In Michelle’s case, various personal life circumstances negatively impacted the course of her EMDR treatment. Collaboration between various team members was therefore essential to keep treatment on track. For instance, one mental health worker was able to help with her daily life issues so that another could make sure that EMDR treatment was provided according to evidence-based guidelines. This collaboration also ensures that treatment is integrated in multiple areas of daily life. This in turn increases the chance that treatment results are consolidated and long-lasting.

Second, the treatment period provided by Youth Flexible ACT teams is limited because of the age limit. Most adolescents in Youth Flexible ACT are between 15 and 22 years, the “transitional age youth” that is preparing to transition to AMHS (2). Although Flexible ACT teams provide a “smooth” transfer to new care providers, clients face several obstacles when moving to AMHS. These obstacles include the erratic organization of service delivery, the different types of care and financial structure, limited access to required services, and a cultural shift in care philosophy (family involvement versus autonomy upon reaching adulthood) (10, 33, 34). Furthermore, a developmental approach focused on the multifaceted age-related needs is often lacking in AMHS services (33). The abovementioned challenges during service transition might cause transitional-age youth to slip through cracks between CAMHS and AMHS and hinder youths’ engagement in care. This, in turn, might lead to potential deterioration of symptoms and relapse (10, 33, 34).

To provide sufficient support when transitioning from intensive Flexible ACT care to less intensive office-based care and/or AMHS, a post Flexible ACT “aftercare” program could be set up in parallel by the same Youth Flexible ACT team. In the United States, a post ACT step-down service had been developed to maintain and extend the progress achieved (35). The adapted ACT offers less intense services with less frequent contact, limited transportation, and reduced case management. Clients with deteriorated functioning are referred back to ACT. Various evaluations have shown that this program is feasible (35, 36). Like the post ACT service, a Youth Flexible ACT “aftercare” program could consist of one or two face-to-face meetings with contacts per month in addition to telephone meetings to help with coordination of services. The coordination of care can then gradually be transferred to, for example, the GP, the social district team, or secondary office-based care. Naturally, improved coordination and cooperation in traditional office-based care and the possibility to provide more outreach care would be a valuable contribution.

Ultimately, the goal is for clients and their family systems to regain control or influence over one’s life, without deterioration and loss of care. However, when a healthy family system cannot take the lead and maintain control, family members may not be able to navigate services independently. In this case, a “resource group” involving care providers, parents, and other important relatives could help. A resource group is a network that can help better navigate critical transitions in life (37). Such a combination of informal and formal networks is already deployed within Adult Flexible ACT and is usually set up right at the start (37). Working with a resource group could ensure continuity of care and prevent the client from falling back on (specialized) mental health care services. The resource group can help identify problems at an early stage and initiate (more intensive) care when needed. This subject deserves further exploration within Youth Flexible ACT.

Michelle’s case illustrates that after years of fragmented care, an integrative, well-coordinated, and flexible care approach offers personally tailored care services that can result in significant improvements. Providing support services in multiple life domains, along with psychiatric treatment coordinated by one multidisciplinary team, helped Michelle move forward. The Youth Flexible ACT model is an example of a care service that can be tailored to the client’s needs and situation and not the other way around. More practical and theoretical knowledge of integrated care approaches is required to further pinpoint essential services for this group of young people with persistent and interrelated psychiatric and social care needs.

## Data Availability Statement

The datasets presented in this article are not readily available due to ethical, legal, and privacy restrictions. Requests to access the datasets should be directed to MB, m.broersen@ggzoostbrabant.nl.

## Ethics Statement

The study involving human participants was reviewed and approved by the Trimbos Ethics Committee. Written informed consent to participate in this Multicenter study was provided by adolescents and parents or legal guardians.

## Author Contributions

RC treated the patient and collected the data. MB wrote the manuscript. NF, DC, HK, and RC read the manuscript and provided the suggestions for improvement. All authors contributed to the article and approved the submitted version.

## Conflict of Interest

The authors declare that the research was conducted in the absence of any commercial or financial relationships that could be construed as a potential conflict of interest.

## Publisher’s Note

All claims expressed in this article are solely those of the authors and do not necessarily represent those of their affiliated organizations, or those of the publisher, the editors and the reviewers. Any product that may be evaluated in this article, or claim that may be made by its manufacturer, is not guaranteed or endorsed by the publisher.

## Abbreviations

ACT: Assertive Community Treatment
AMHS: Adult Mental Health Services
CCAF: Centre for Certification ACT and Flexible ACT
CAMHS: Child and Adolescent Mental Health Services
DSM: Diagnostic and Statistical Manual of Mental Disorders
EMDR: Eye Movement Desensitization and Reprocessing
ERT: Emotion Regulation Therapy
F-ACT: Flexible Assertive Community Treatment
GP: General Practitioner
IPS: Individual Placement and Support
OQ-45: Outcome Questionnaire 45
PIH: Psychiatric Intensive Homecare
PMT: Psychomotor Therapy
PNP: Psychiatric Nurse Practitioner
PSS-SR: PTSD Symptom Scale – Self-Report
PTSD: Post-Traumatic Stress Disorder
SFT: Systemic Family Therapy
STROBE: Strengthening the reporting of observational studies in epidemiology
USA: United States of America.
